# Recent Developments and Applications of Drone Swarm: Techniques, Strategies, and Challenges

**DOI:** 10.3390/s26102943

**Published:** 2026-05-08

**Authors:** Ravi Raj, Andrzej Kos

**Affiliations:** 1 Department of Mining, Industrial and ICT Engineering (EMIT), Manresa School of Engineering (EPSEM), Universitat Politècnica de Catalunya (UPC), BarcelonaTech, 08242 Manresa, Barcelona, Spain; 2 Faculty of Computer Science, Electronics, and Telecommunications, AGH University of Krakow, Aleja Adama Mickiewicza 30, 30-059 Krakow, Poland; kos@agh.edu.pl

**Keywords:** artificial intelligence (AI), deep learning (DL), drones, swarm intelligence, unmanned aerial vehicles (UAV)

## Abstract

The dynamic and complex environment, together with challenging assignments, requires that unmanned aerial vehicle (UAV) systems evolve toward cooperation, autonomy, and cognition. UAV swarms illustrate a revolutionary development in aerial robotics, which utilizes coordinated autonomy to improve operational efficiency. This study offers a detailed examination of UAV swarm systems, the latest developments, and their different applications. The main domains, such as intelligent path planning, work allocation, coordinated control, and safety issues, are analyzed, focusing on the integration of Artificial Intelligence (AI) and Deep Learning (DL) to enhance decision-making and agility. We address the constraints and potential advances in the field of swarm intelligence to facilitate additional research endeavors. The ongoing advancement of drone swarm technologies and its exploration of military uses highlight the increasing importance of anti-drone swarm strategies. Therefore, studying these strategies will have substantial practical importance in preventing and countering drone swarm combat. Thus, this article provides detailed drone swarm applications and the importance of anti-drone swarm techniques in strategic operations. Furthermore, this comprehensive study of the literature aims to offer innovative perspectives on the latest advances in UAV swarm intelligence technology. Future research trends and challenges are discussed to find the research gap.

## 1. Introduction

Today, there is an emerging need for aerial drones with various functions for civilian and military purposes. There is considerable interest in the advancement of innovative drones capable of independently navigating diverse environments and executing numerous missions [1]. Over the past decade, the wide range of uses for these drones has garnered significant interest, resulting in the development of many types of drones with diverse sizes and weights. Drones are aerial robots that include UAVs capable of navigating thousands of kilometers, as well as smaller drones designed for operation in limited environments [2]. Drones are classified as aerial vehicles that operate without a human operator, either manually or independently, and transport military or peaceful payloads. Ballistic or semi-ballistic vehicles, cruise missiles, howitzer weapons, submarine munitions, and satellites are not recognized as drones. Progress in manufacturing, navigation, remote control technologies, and energy storage systems has enabled the creation of various drones that can be employed in scenarios where human presence is challenging, unfeasible, or harmful [3].

**Figure 1:**
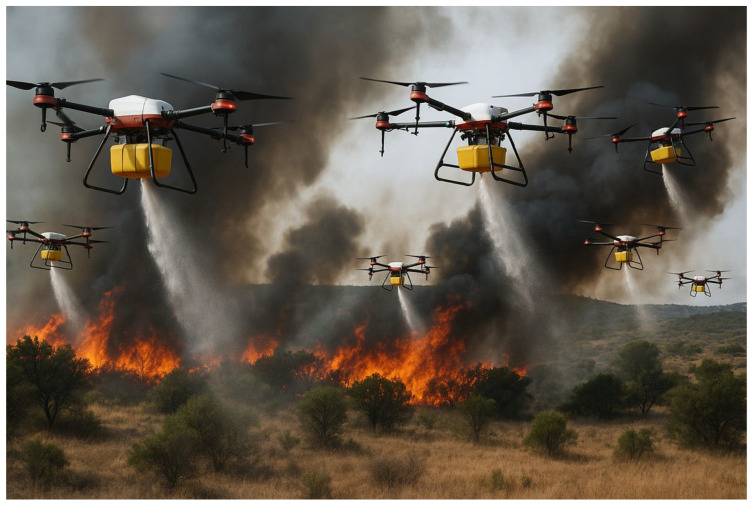
Swarm of drones for wildfire management [5].

Drones fitted with various sensors capture environmental data in real time, later consolidating and transmitting such data to the operational center, enabling operators to more effectively assimilate on-site information and implement relevant steps. The drone swarm, characterized by a high degree of autonomy, can operate independently of human control and perform tasks through collaborative efforts. A further important benefit of drone swarms is their ability to scale up efficiently to do more complex jobs. Through the distribution of responsibilities and loads to numerous individual drones, the drone swarm will do tasks concurrently, hence minimizing processing time and enhancing fault tolerance. The restricted dimensions of the nodes enable the drone swarm to be challenging to identify, hence enhancing its survival in strategic environments. Each drone within a swarm is configured only with select operational hardware or sensors. The information exchange demands that the drone swarm system be fully interconnected, which is essential for the accurate execution of the mission. However, the drone swarm encounters predominantly highly complex, severe, and intensely adversarial contexts. The small structure in a drone swarm is more prone to malfunction than standard drones, resulting in an unreliable fully integrated network within the swarm [4]. Figure 1 provides an exemplary depiction of a swarm of drones employed to manage wildfires.

**Figure 2:**
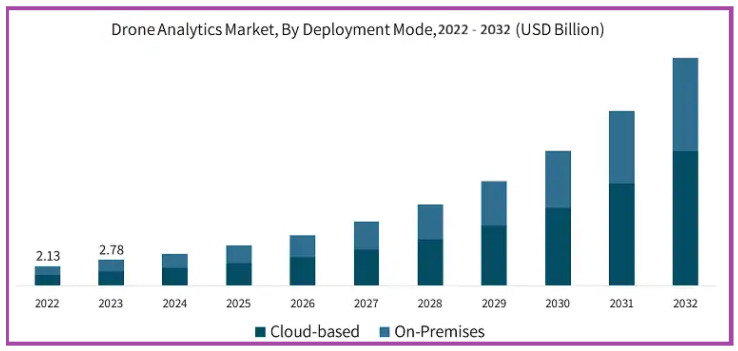
Forecast of the drone market size by deployment mode [11].

The dynamic and complex environment, together with difficult missions, demands the evolution of UAV systems towards clustering, self-reliance, and cognition. Drone swarms frequently exhibit collaborative behavior to function as an intelligent cooperative and complete tasks that would be impossible or impractical for normal people to do alone [6,7]. Individual drones in the swarm will recognize their immediate surroundings and respond appropriately, with or without explicit understanding of the swarm’s broader goal. The developments in drones for swarms have presented revolutionary possibilities represented by drone swarms. The swarms utilize aerial transportation, rapid agility, and broad visibility features, making them essential in various applications [8,9]. These technologies have been applied for observation and environmental mapping, airbase communication networking, target identification, building monitoring, and construction, as well as load transportation and shipment, proving especially advantageous in locations that humans cannot access, including jungles and disaster-stricken areas [10]. Despite their capability to function on the ground, in the water, in the air, and in space, drone swarms are inherently reliant on information. The universal factor among all swarms is the necessity of maintaining steady communication connections among drones and to ensure that the information is analyzed effectively and suitably. Swarms are defined as many unmanned systems that may coordinate their actions to achieve common objectives. Drones are poised to significantly alter our methods of work, lifestyle, and exploration of the surrounding environment. Figure 2, created by Global Market Insights [11], illustrates the past, present, and future projection of the size of the drone market by types of deployment modes.

A multitude of uncoordinated unoccupied systems does not constitute a “swarm”; it represents a flood. A swarm has various components that synchronize and adjust their movements to create a unified, emergent entity [12]. A wolf pack differs significantly from a simple assemblage of wolves. Ant colonies are capable of building structures and engaging in warfare; yet, a multitude of disorganized ants is incapable of achieving either task [13]. To fully unlock the benefits of the robotic revolution, we need to create robotic systems that can work together and coordinate with human operators to move and fight effectively in combat situations. Swarms in nature are entirely emergent phenomena that result from fundamental principles. Although they lack individual intelligence, their colonies can demonstrate remarkably intricate behavior. They can quickly and effectively search for food and determine the ideal way to transport it back to their nests. Bees can “vote” on new nesting sites, collectively determining the most advantageous spots. Ants can eliminate and transport substantial prey through collective cooperation. Termites build substantial edifices, whereas ants create bridges or buoyant formations across water utilizing their bodies. The frog then resides within the colony during the arid season, benefiting from the nest’s humidity and protection from predators [14].

In contrast, “slave-making” ants employ a tactic that deceives other ants into laboring for their colony. Slave-making ants invade rival ant colonies to abduct their larvae, transporting them to their nest, where they are nurtured to serve the slave-making ants’ workers [15]. The abducted ants, having spent their entire lives in a competing colony, are oblivious to the fact that they have been seized by an opposing species. The slave-making ant Polyergus breviceps advances this strategy by not only plundering larvae but also commandeering a complete colony. A Polyergus queen can penetrate a competing colony, eliminate the reigning queen, and assume the role of the new queen. The commandeered colony and its laborers then nurture their progeny [16]. These instances of animals that use communication signals within a swarm are akin to spoofing and cyber-attacks in the strategic sector. Swarm security—ensuring the trustworthiness of other swarm members, particularly those in leadership roles—will be very crucial for drone swarms. The abilities of drone swarms, enhanced by modern sensors and sophisticated control systems, are becoming crucial and widespread in real-world uses. Drones are increasingly influencing several industries and scientific disciplines.

### 1.1. Swarm Definition

The term “swarm” suffers from a lack of a standardized definition, despite its prevalent use in both conventional and technical environments, including the administrative and civilian sectors. The difficulty involved in defining “swarm” is exacerbated by its linguistic characteristics, particularly its dual function as both a noun and a verb, resulting in conceptual uncertainty and associated interpretative challenges. To address this issue, it is crucial to differentiate between the noun and verb forms and to specifically confine the current discussion to the noun variety. This approach enables the exclusion of the features and implications linked to the verb form, including swarming, swarm actions, and swarm cognition, which frequently suggest flexible and adaptable procedures as well as concepts of independence, autonomy, and various personality characteristics. This exclusion facilitates a more accurate and nuanced definition of a “swarm” as a noun, free from the superfluous connotations and meanings often associated with the verb form. The significance of formulating a precise and unambiguous explanation of the noun form is paramount. It is crucial to promote effective communication, collaboration, and development among participants, such as scientists, programmers, and users. A consistent definition of “swarm” will create a single terminology and system for comprehension, facilitating the creation of an integrated comprehension and a coherent strategy for swarm capabilities development. A clear and universally recognized definition would establish a framework for the advancement of swarm-related principles, doctrines, and techniques and would allow the assessment of the efficiency and value of swarm skills in many situations. Establishing a “swarm” as a noun is a crucial prerequisite to building a powerful and efficient swarm capacity, which is needed to realize the full value of swarm advances in favor of national security interests [17]. The proposed definition by [17] for the term “swarm” is as follows: an assembly that functions collaboratively under the directives of a singular operator via a shared architecture.

### 1.2. Types of Swarm

**Figure 3:**
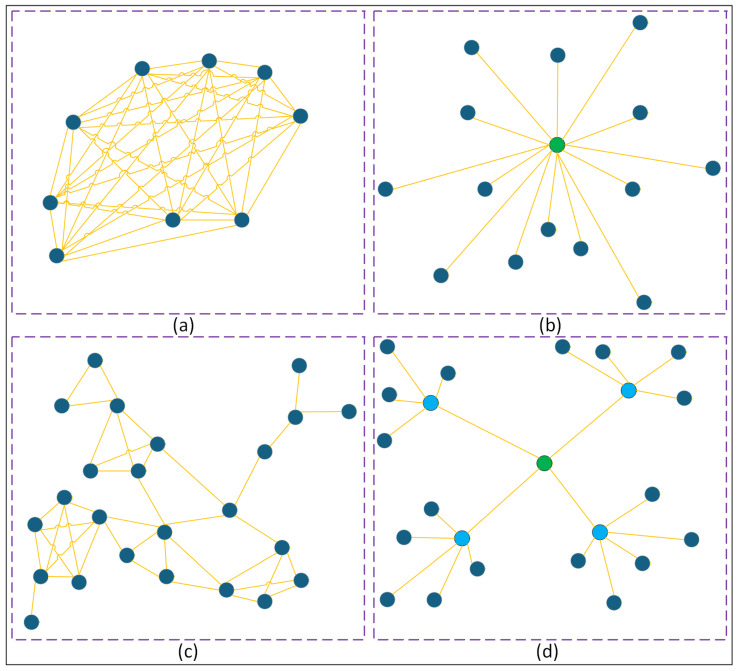
Depiction of different types of swarm command and control models [20]. Where, (**a**) Coordination by consensus, (**b**) Centralized control, (**c**) Emergent coordination, and (**d**) Hierarchical control.

Scaling multi-vehicle operation to accommodate massive swarms requires significant transformations in the command-and-control framework. The Naval Postgraduate School is developing a 50-on-50 swarm versus swarm aerial duel, while researchers at Harvard have constructed a swarm of more than 1000 basic robots collaborating to form simple configurations [18]. As the quantity of elements in a swarm escalates, human oversight must progressively transition to collective control of the swarm rather than the supervision of each individual piece. The effective command-and-control in a swarm is a developing field of research. The potential command and control frameworks are encompassed, arranged from greater centralization to progressively dispersed authority [19]. Figure 3 provides a description of different types of command and control models of swarms.

#### 1.2.1. Coordination by Consensus

Swarm objects interact and settle on an approach using voting or auction-type mechanisms. An illustration of this type of swarm example is provided in Figure 3a.

#### 1.2.2. Centralized Control

Swarm members communicate information to a central controller, which subsequently assigns duties to each member separately. The swarm elements interact with a centralized controller that organizes all tasks. An illustration of this type of swarm example is given in Figure 3b.

#### 1.2.3. Emergent Coordination

Coordination emerges naturally as various components of the swarm respond to each other, such as in animal swarms. This type of swarm is illustrated in Figure 3c.

#### 1.2.4. Hierarchical Control

Individual swarm components are governed by “squad”-type agents, which are subsequently managed by superior controllers, and so on. This type of swarm is illustrated in Figure 3d.

Each model has its strengths, and the best one to use may depend on the situation. Although completely decentralized swarms can find the best solutions for complicated problems, similar to how ant colonies pick the quickest path to bring food home, it might take several attempts to get to the best answer, which requires time. Centralized or hierarchical strategies can help swarms quickly find the best or a good solution, but they require more bandwidth to send data to a central point that then sends instructions back to the swarm. In scenarios with poor bandwidth communication, swarm units may employ consensus-driven action through voting or auction processes [21]. In the absence of direct communication, swarm units might still depend on indirect communication to achieve adaptive coordination. It can occur through interaction, similar to the behavior of animals that flock or herd, or through reactive interaction that modifies the surroundings.

The remaining article has been divided into five parts, including Section 2, which provides the literature survey; Section 3, which explains different applications of drone swarms; Section 4, which describes different algorithms used in drone swarms; Section 5, which discusses future research trends and challenges; and Section 6, which provides the conclusion.

## 2. Literature Survey

AI-driven drone swarms are rapidly transforming the dynamics of aerial warfare, reallocating military superiority from costly assets to synchronized autonomy. AI now enables numerous low-cost drones to function as an integrated system rather than as separate entities. These swarms can surpass conventional air defenses, react instantly, and carry out operations without incurring losses. AI drone swarms have emerged as a highly unpredictable force in modern combat, while major countries compete to deploy them.

### 2.1. Trajectory Generation for a Swarm of Drones

Trajectory generation is a primary task for the swarm of drones whenever it is deployed for certain tasks. An innovative method for creating optimal path times for a swarm of quadrotors to navigate through predetermined checkpoints while optimizing maneuverability and avoiding collisions is defined in [22] and simulations and experimental flight tests demonstrate that the suggested method can create optimized aggressive trajectories for a swarm of automated racer drones. Aerial surveillance with multiple UAVs possesses great potential in various applications. However, current research on swarm tracking generally fails to provide sustained target visibility in obstructed situations. To rectify this shortcoming, ref. [23] proposes a decentralized planner that optimizes target visibility while guaranteeing collision-free moves for swarm tracking. This study first looks at how well each drone can track using a decentralized aerodynamic exploration front-end, which creates the best path to ensure safe flying areas and visible zones. A spatial–temporal optimizer subsequently produces a polynomial trajectory that adheres to the corridor restrictions. Performance objectives also involve collisions between vehicles and interference prevention. Ref. [24] examines the issue of efficiently distributing operations and creating collision-free paths for drone swarms to function in environments packed with obstacles. The planned Swarm Allocation and Route Generation (SARG) system combines optimal assignment of tasks with dynamic viable route planning, facilitating rapid mission execution while maintaining safe navigation across complex 3D environments. The system leverages quadrotors as a primary prototype system, integrating both drone-to-obstacle and drone-to-drone collision-avoiding algorithms, along with an optimized path planning algorithm that improves the performance of concurrent graph searches.

Ref. [25] examined the attributes and properties of many swarm optimization techniques, including ant colony optimization (ACO), fruit fly optimization algorithm (FOA), artificial bee colony (ABC), and particle swarm optimization (PSO). This study offers a detailed assessment of the most significant research on algorithms for planning drone trajectories. This article focused on evaluating the influence of the swarm algorithm and its effectiveness in drone trajectory prediction. The study talks about a popular algorithm and its models designed to improve drone paths using swarm intelligence and how it affects the best routes drones need to take. Ref. [26] introduces LEVIOSA, an innovative framework to generate trajectories of UAVs based on input of audio and text. The system utilizes multimodal large language models (LLMs) to comprehend natural language commands, transforming text and voice input into actionable flight routes for UAV swarms. The method seeks to streamline the intricate process of multi-UAV trajectory development, which has substantial applications in areas including rescue operations, agriculture, building monitoring, and entertainment. The system includes two main new ideas: a way to evaluate how reliable flight paths are based on several criteria and a structured approach to improve task performance. The improvements guarantee adherence to the user objectives. The framework uses several multimodal LLMs for better planning, turning natural language instructions into 3D points that guide UAV movements and help each UAV’s basic controller follow its specific 3D path as part of the overall plan.

### 2.2. Trajectory Prediction for the Drone Swarm

Trajectory predictions for the drone swarm during swarm-to-swarm military warfare and transport are the most important technique that can enable drone effectiveness. Ref. [27] begins by delineating measurements of link channel capacity to document data exchange within UAV swarms, thus establishing a framework for a UAV swarm system. The paper presents a dynamic graph neural network (DynGN) model that uses a structure with an encoder and decoder, combining a graph convolutional network with a gated recurrent unit. This method looks at both the changing network setup and the movement data of UAV swarms at the same time, which helps improve predictions based on experiments that emphasize prediction accuracy, node number stability, and noise resistance to evaluate the model’s performance. The results demonstrate that the DynGN model surpasses traditional trajectory prediction models, attaining significant enhancements in precision and fitability. Furthermore, its resistance to noise in dynamic trajectory data underscores its significant applicability in real-world mission scenarios. Ref. [28] introduces a system that helps drones predict where other drones will go is introduced, making it easier for them to work together in a noisy environment. The suggested method allows each UAV to anticipate the intended trajectories of other drones in settings void of interaction. The anticipated intended trajectories will function as input for a distributed model predictive control (DMPC) methodology. The suggested technique combines (1) a trajectory prediction model that uses EvolveGCN, a type of Graph Convolutional Network (GCN) good at handling changing graphs, and (2) a training method that uses KKT scenarios to teach the neural network about DMPC. The results show that the proposed method outperforms leading benchmarks, providing nearly optimal control effectiveness without interaction.

Ref. [29] applies a transformer-based self-attention technique to a graph attention convolutional network architecture. During the encoding step, a graph convolutional neural network (GCN) is utilized to enhance the depiction of temporal data. Thereafter, an attention-guided graph neural network is employed to proficiently record and encode communication characteristics among drones in the swarm. The transformer focus technique is subsequently incorporated into the decoder to enhance the prediction method. This encoder–decoder setup is made to capture and understand the timing and location of how each drone communicates, leading to accurate predictions of their paths that consider the unique actions of drone swarms. Experimental findings on an infrared dataset for estimating drone swarm trajectories, along with the publicly accessible pedestrian trajectory estimation datasets ETH/UCY, indicate that the suggested strategy surpasses traditional techniques. Ref. [30] addresses the challenges of trajectory prediction and intention recognition for UAV swarms in the field of defense and investigates a novel approach for UAV swarm trajectory estimation by automatically developing a UAV swarm dataset and employing a Long Short-Term Memory (LSTM) network for experimental verification. Traditional research has primarily focused on evaluating the trajectories of individual UAVs; however, the dynamic behavioral patterns of UAV swarms have not been investigated yet. This technique produces a UAV swarm data set comprising several types of flight and uses the LSTM model to simulate and predict their movements. The experimental findings demonstrate that LSTM can proficiently represent the temporal dynamics of UAV swarms, anticipate their subsequent directions, and determine expected interference intentions, hence offering early warning assistance for air defense systems. This study presents a novel technical concept to identify the intent of the UAV swarm and advances the academic trajectory of developing advanced air defense technology.

### 2.3. Techniques to Encounter the Swarm of Drones

During strategic warfare, one of the major issues nowadays is battling a swarm of drones coming from the enemy. Today, many researchers are working to develop an optimized technique to counter the swarm of drones during combat warfare. Ref. [31] discusses the decision-making processes involved in UAV swarm-against-swarm offense/defense confrontations while targeting aircraft carriers in open waters. The system is designed as a multi-agent framework. Each UAV is distinctly modeled as an autonomous entity, able to make decisions informed by the swarm’s operational rules, tracking radius, and enemy positioning. A distributed auction-based algorithm is developed for swarm UAVs, constrained by an inadequate data-sharing rate. A critical aspect of the suggested technique is its capacity, which allows each UAV to make real-time decisions throughout flight, despite the constraints of restricted communication hardware regarding the update rate within the UAV interaction system. The algorithm variables are optimized to achieve enhanced target distribution and elimination. Ref. [32] examines a dynamic swarm versus swarm UAV battle scenario and introduces an autonomous offense-defense confrontation decision-making (ODCDM) system. The ODCDM algorithm uses a system in which each UAV acts as an individual unit that can solve its own decision problems by sharing information with nearby units. In each decision-making phase, the swarm strives to identify an effective target distribution strategy, while each UAV then adopts the relevant behavioral protocols, resulting in innovative offensive and defensive actions. The decision-making process during an offense-defense conflict can be classified into target distribution choices through a consensus-based auction algorithm (CBAA) and social-force-based swarm mobility decisions. An offense-defense strategy is included in the objective distribution optimization model, giving UAVs the option to choose between a stronger offensive or defensive position. A combat stimulus is deemed to propel UAVs toward the designated target, grounded in the fundamental collective actions of collaboration, division, and coordination.

An optimal solution is examined in [33] for the deployment of air defense systems versus drone surveillance swarms. The objective is to establish the placement of various air defense systems within the specified region, thus maximizing the cost of enemy drones traversing that sector. The expense is determined by a counterpart drone trajectory optimization issue. The study offers a detailed method for solving smaller problems and a special technique for larger ones to tackle this challenging situation. This study employs an evolutionary architecture that uses six prominent evolutionary algorithms. The capacity of various human and unmanned aircraft technologies to collaboratively confront and neutralize an airborne threat is crucial in contemporary conflict contexts. The study in [34] presents essential ways to provide collaborative threat engagement across numerous interconnected agents, including a swarm of combat-capable drones with interaction abilities. This project integrates AI-driven decision-making and control methodologies for a swarm of loyal wingman drones to orchestrate effective military operations collaboratively and autonomously. This study applies these ideas in a combat situation meant to test the loyal wingman strategy, which is considered a strong option for working together on decisions and managing tasks with minimal oversight.

### 2.4. Optimal Intelligence of the Swarm of Drones

The swarm of drones needs to have an optimal intelligence level to get involved in higher-risk applications, including firefighting, disaster support equipment, and many more. Ref. [35] examines the effectiveness and efficiency of popular swarm intelligence techniques, such as the Cuckoo Search (CS), Grey Wolf Optimization (GWO), Elephant Herd Optimization (EHO), Salp Swarm Algorithm (SSA), Monarch Butterfly Optimization (MBO), and Particle Swarm Optimization (PSO), in resolving sixth-generation (6G) mobile networks that face a number of issues due to the development of future-generation Internet-of-Things (IoT) services, including huge connectivity, greater network bandwidth, and exceptionally low latency. This study specifically examines how three scenarios function when various swarm intelligence techniques are present. Ref. [36] provides a novel, robust guidance control approach for swarms of drones. This model creates a swarm resilience mechanism that dynamically combines swarm cohesiveness with personal integrating characteristics by using a stochastic transition method for interacting patterns based on a Markov decision process. This method makes it possible for knowledgeable agents to successfully lead people outside of their initial topological communication range. Additionally, the guidance control issue is presented as a problem with multiple objectives that uses a flexible optimization technique to balance motion uniformity and system adaptability. Even with just 5% knowledgeable agents, thorough simulations show that the framework can direct a swarm of 1000 drones to complete complicated routes with a 99.95% accuracy rate. Experiments conducted in the physical world with Crazyflie micro quadrotors confirm the concept’s usefulness.

Ref. [37] provides a method of drone swarm choreographing that integrates large language models (LLM) with reliable motion control. SwarmGPT makes it possible for beginners to create and improve performance on songs by utilizing the reasoning powers of LLMs and an easy-to-use interface. By slightly modifying risky trajectories, an optimization-based security filtering guarantees viability. The suggested method is verified in real-world trials with up to 20 drones as well as in simulation with up to two hundred drones, showing adaptability for various swarm dimensions and musical genres. Motion fundamentals minimize planning mistakes and aid with visual understanding, but they constrain practical versatility. Finally, SwarmGPT provides a guide for securely incorporating LLMs into complex robotic platforms and paves the way for more extensive uses, like non-professional swarm control in rescue missions and emergency management. Ref. [38] presents a new paradigm for Physics-Regularized Adaptive Control (PRAC) that uses neural back-stepping. This study uses Radial Basis Function Neural Networks (RBFNNs) as a structural underpinning to construct innovative physics-regularized neural networks (PRNNs), which are key to PRAC. The PRAC approach incorporates physical prerequisites, including equilibrium requirements, system resiliency, and energy loss standards, into the web-based adaptable rules of the PRNNs as distinct regularity factors rather than viewing the neural network as a straightforward approximator. The learning procedure becomes a Lyapunov-guided restricted optimization as a result of the gradients of such terms effectively constraining the PRNN weight adaptation. This improves control accuracy and increases the acquired dynamics physical stability and comprehensibility.

## 3. Some Major Applications of Swarm of Drones

**Figure 4:**
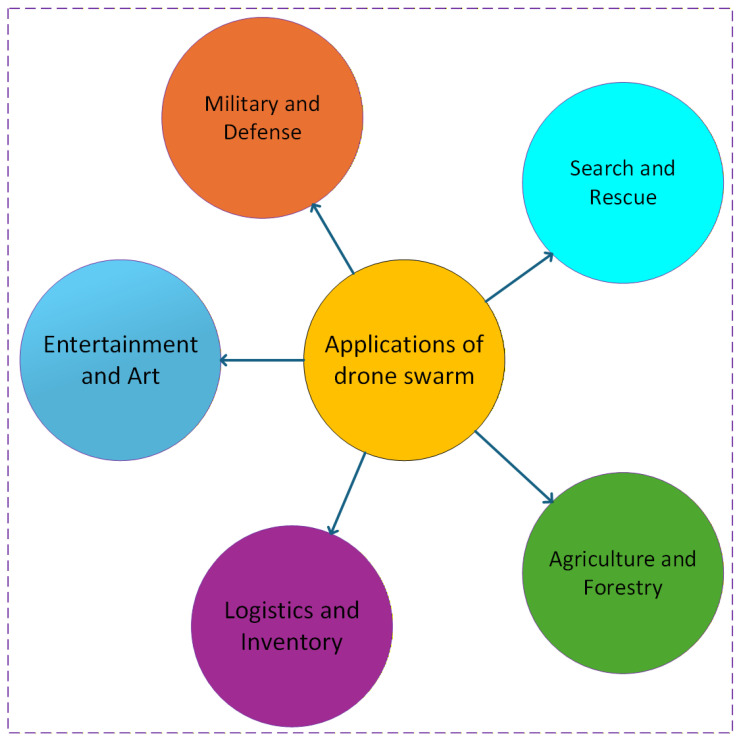
Different applications of a swarm of drones.

Drone swarms have significantly changed the cost-benefit evaluation in the field of defense. In clear contrast, portable drone swarms present a cost-effective, adaptable, and robust option. Swarms provide remarkable situational awareness and agility in real time by enabling coordinated operations across land, air, and sea. It can destroy defensive systems, infiltrate enemy infrastructure, and carry out operations by deploying both stealth and physical force while simultaneously exhibiting significant tolerance for casualties. Despite extensive research on single-UAV equipment and their existing uses, constraints remained that might be addressed through the use of a swarm of drones, which are examined in this part. Figure 4 provides a list of different applications of a swarm of drones.

Drone swarms offer enhanced benefits by covering significantly greater areas in shorter time frames. With built-in intelligence, UAVs can independently collect information on things they see and recognize while also working together as a group to improve surveillance tasks, such as covering the best areas within the limits of vehicles and sensors [39,40], ensuring the safety of land vehicle convoys [41], and providing ongoing monitoring [42,43]. Various technologies have been created to offer counter-drone systems capable of detecting unwanted drone activities and implementing mitigation strategies. A drone defense system typically has two primary subsystems: UAV detecting and monitoring and neutralization or mitigation systems. Recognition and monitoring can be performed using specific sensor methods, such as radio frequency (RF) tracking and spoofing, thermal and RGB cameras, RADAR, and electromagnetic sensors, or by combining these technologies to improve the accuracy of monitoring and detection [44].

**Figure 5:**
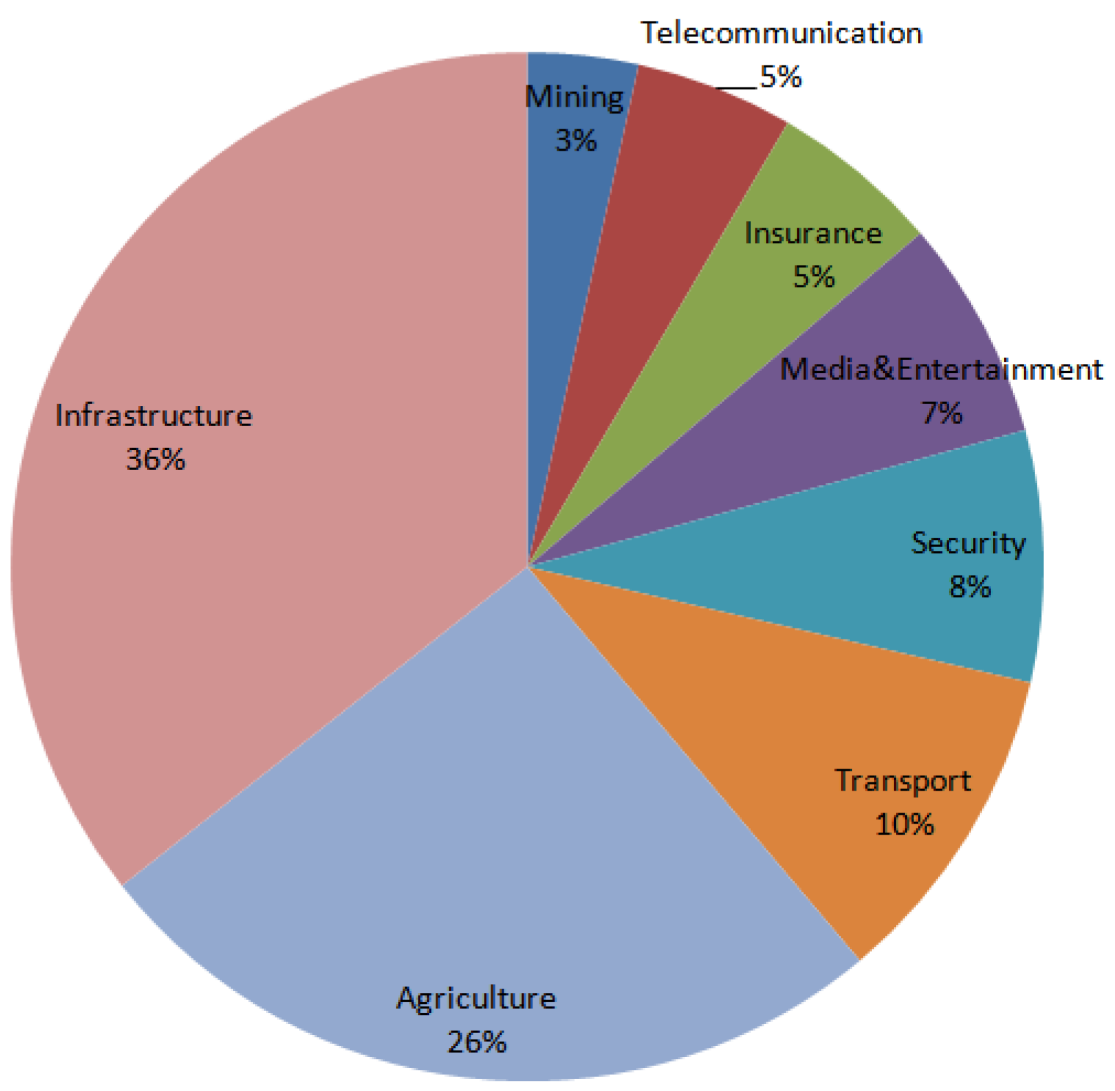
Illustration of the quantity of drones by application area in different sectors [49].

The past few years have witnessed a substantial transformation in monitoring the environment, mainly attributable to advances in drone technology. Historically, environmental monitoring employed a blend of conventional techniques, including manual sampling and terrestrial sensors, alongside expensive piloted planes. Using drones, or UAVs, researchers can gather precise high-quality data more rapidly and economically than previously achievable for environmental data collection [45]. UAVs have facilitated the assessment of coastal erosion and the identification of wildlife movement patterns, allowing scientists, organizations, and authorities to monitor and understand the environment in innovative ways. Using clusters of UAVs in ecological monitoring activities presents significant advantages. The authors in [46] introduced a multi-drone platform for real-time flooding monitoring and surveillance, a typically imprecise task when utilizing standard forecasting approaches. Each UAVs is equipped with a collection of portable sensors that might be transported by flood currents, facilitating communication with UAVs to assess the pattern and velocity of the flood. More studies related to environmental monitoring are available, including the evaluation of the level of pollution and the assessment of forest ecosystems [47,48]. Figure 5 illustrates the percentage of application of various types of drone applications.

Aerial drone displays are considered one of the most effective entertainment uses of UAV swarms. In 2016, Intel established the Guinness World Record for the largest number of UAVs airborne together with an arrangement of 100 drones featuring LEDs [50], subsequently achieving a further record of 500 drones in a single year [51]. Ehang, a Chinese company, set new records by executing a light display using 1000 UAVs in 2017 [52], subsequently achieving another milestone using 1374 UAVs in the same year [53]. The most recent record for the largest drone display was set by Intel in 2018, using 2018 UAVs [54]. In these functions, every drone typically consists of a basic UAV framework (e.g., a quadcopter) outfitted using an integrated flight controller, GPS sensors for locating, customized LEDs, and an interface for interaction with a base station. The base station is used to estimate the specific missions necessary for all UAVs throughout the event. Subsequently, each mission is transmitted to the respective drone, where it is conducted by the integrated flight controller. The base station consistently oversees the swarm’s state throughout the performance and facilitates controls for any necessary emergency situations.

**Figure 6:**
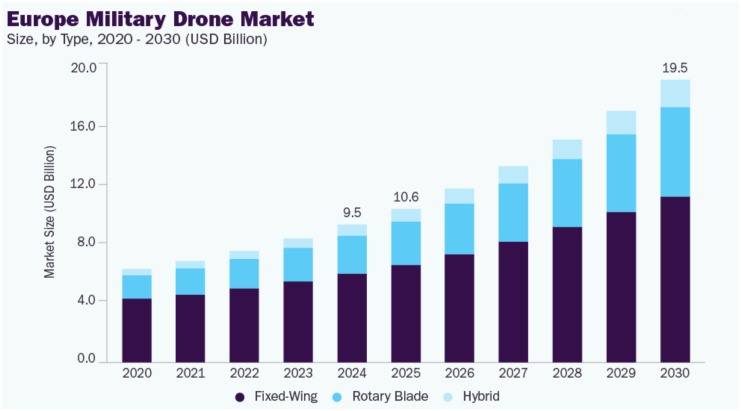
Forecast of the European military drone market [55].

In recent times, drones have transformed the domains of security and the surveillance. The amalgamation of sophisticated technology with adaptable unmanned systems has initiated a novel epoch for surveillance, intelligence, missions, and logistics. Autonomous drones are leading this revolution, providing improved monitoring and security skills. Drones using modern technology, such as advanced electronics and enhanced software, are set to deeply impact the defense industry, reflecting progress observed in commercial markets. Recently, Europe has been under threat due to the ongoing Russian and Ukrainian conflict; therefore, it is expected that the market for military drones in Europe will increase sharply in the coming years. Figure 6, created by Grand View Research [55], illustrates the past, present, and future projection of the size of the European military drone market by types of drones.

Search and rescue (SAR) operations are a category of emergency job. One of their responsibilities is to rescue people in difficult circumstances, such as emergencies or natural disasters. Currently, professionals, volunteers, and aerial assets are mainly involved in these activities. Nevertheless, this strategy has drawbacks: extensive coverage of the region requires a substantial workforce, and the deployment of helicopters (or other types of search and rescue aircraft) incurs significant financial expenses [56]. Consequently, the necessity for investigating alternative methods for conducting SAR operations arises; these innovative ideas may decrease both costs and duration while preserving the standard of SAR operations. Drones have recently emerged as an invaluable technology for rescue teams tasked with locating individuals in expansive and challenging terrains. These drones are more rapid and economical to deploy than conventional piloted planes. Although a single drone can assist personnel by quickly providing an aerial perspective, future developments may allow numerous drones to collaborate as a swarm, thus reducing the time required to identify an individual [57]. In SAR operations, drone swarms can play a vital role in dense forests and flood situations. Drones are useful in different sectors, including military warfare. The significant benefits of drones have led to numerous studies aimed at optimizing and enhancing their performance.

In summary, drones are becoming more and more crucial in recent times. They have successfully identified their niche in the demanding and technical fields of defense, agricultural uses, logistics, supply chain management, and security and surveillance. This advanced equipment has also discovered its elegant applications. In domains such as homemade crafts, both beginner and commercial photography, racing tracks, and drone athletics, etc. Recently, one of the most impressive applications of drones has been swarm drone displays. Whether at community celebrations or national events, swarm drone displays are a captivating spectacle. Programs of this nature are currently in their infancy and possess significant potential. Furthermore, they are environmentally sustainable in comparison to firecrackers. Drones, owing to their compact dimensions and exceptional dexterity, offer extensive coverage of vast areas. This allows the user to obtain a comprehensive holographic representation of the entire area. The potential for integrating drones with the virtual world is virtually limitless and would significantly enhance various applications. To leverage these capabilities, both amateurs and industry professionals are devising methods to integrate drones within their surroundings, significantly transforming numerous organizations and generating new opportunities. Effective implementation of technology in UAVs can ease several emergency scenarios in both civilian and military contexts. The deployment of drones can facilitate the delivery of emergency healthcare supplies, regardless of transportation limitations [58].

## 4. Algorithms for Autonomy of Swarm of Drones

We appear to be on the edge of an AI-driven transformation in strategic operations. Researchers have examined various aspects of this transformation, although one of the most dynamic discussions refers to the issue of lethal autonomous weaponry [59]. Some researchers believe that autonomous weapons, particularly autonomous drone swarms, have the potential to dominate the battlefield due to their ability to operate with minimal human intervention, execute complex strategies, and respond rapidly to changing combat conditions. Enhanced autonomy can facilitate improved survivability. A multitude of human-operated drones would be susceptible to the loss of the operator. In a swarm of human-operated drones, the operator represents the most vulnerable element, as neutralizing or disabling them would render the swarm inoperative. A human controller may potentially suffer from illness or injury regardless of enemy engagement.

A completely autonomous drone swarm does not encounter such risks. Enhanced autonomy enables a drone swarm to accelerate decision-making. In the scenario of a remotely operated drone swarm, a controller must obtain data from the field drones, analyze these data, determine the deployment of sensors or armaments, and execute the command to engage targets [60]. The delay may cause the adversary to initiate fire, alter their position, or take alternative defensive measures. The delay will increase with a larger swarm of drones since the operator can focus on another site. Delegating decision-making to AI in the field can expedite the decision-making process, hence enhancing the swarm’s survivability and capacity to inflict damage. Enhanced autonomy facilitates the novel deployment of a drone swarm. It can be designed to execute several attacks over an extended duration, interspersed between assaults, allowing for strategic planning and minimizing adversary detection [61].

**Figure 7:**
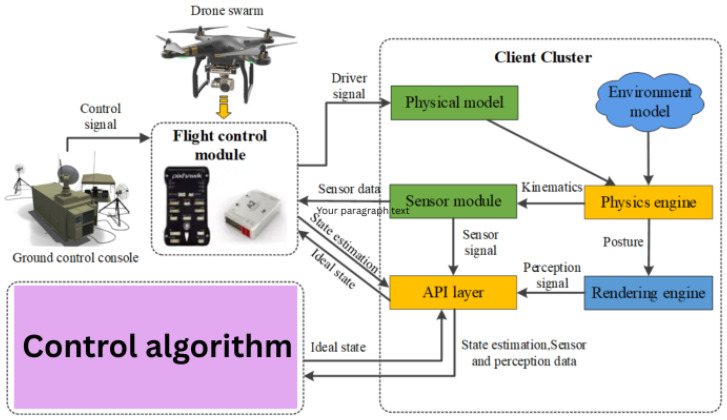
The control algorithm-based swarm patrolling [62].

Currently, various types of drones have emerged due to advancements in the smaller size of electronic parts, including microprocessors, batteries, sensors, and navigation systems. Increased autonomy in drone swarms facilitates better control. Autonomy enables numerous drones in a swarm to adhere to a singular leader, maintain consistent distances from one another, evade obstacles, and execute assaults on targets. Each automated function reduces the number of tasks that require operator intervention, and intricate swarms of drones impose heightened cognitive demands on human controllers, as they require the ability to process and respond to a larger volume of information and coordinate multiple drones simultaneously. Extensive swarms require increased operating demands and additional sensors to transmit data to operators, which can lead to operator fatigue and potential errors in decision-making due to the overwhelming amount of information being processed. Tang et al. [62] present an algorithm for the swarm patrolling tasks, which is illustrated in Figure 7.

Numerous studies have concentrated on enhancing the autonomy and collaborative capacities of UAVs in complex situations. To solve the problem of communication loss in drones, Fei et al. [63] created a system that recognizes obstacles using a deep Q network combined with a Faster R-CNN model and a data storage method. Experimental findings indicate that the agent trained using the suggested algorithm model possesses the capability to autonomously navigate and avoid collisions. Milias et al. [64] presented a vehicle-mounted radar device for drone detection. Experimental data indicate that the proposed vehicle-mounted radar can detect drones at distances of up to 440 and 500 m, respectively. Prabakar et al. [65] investigated the task shift and directional control capabilities in the interaction between the UAV and the Internet of Things power source, highlighting their critical importance in improving UAV operations, capital efficiency, energy efficiency, and real-time decision-making. Jones et al. [66] investigated the potential applications of UAV in high-risk or high-cost situations, particularly the possibility of autonomous UAV swarms working collaboratively to perform tasks. They identified challenges in environmental modeling and path planning and examined future research directions and unresolved issues. Using real-time detection from aerial RGB (Red, Green, Blue) cameras, Horyna et al. [67] developed a decentralized swarm navigation approach to direct UAVs towards potential objects of interest. The effectiveness of this method in detecting objects of interest, determining the difference between real and false positives, and helping with joint environmental exploration was tested using simulations and real-world studies.

From a doctrinal perspective, a drone swarm might be deployed for many military missions. Initially, it will guarantee a scattered allocation of sensors tasked with reconnaissance, observation, tracking, exact location, and tracing. This task can be accomplished both actively and passively. For example, many widely placed sensors can find sources of signals by looking at differences in arrival times and frequencies caused by the Doppler effect due to movement [68]. In active recognition, distributed sensors work like a multi-static radar system, where one sensor sends out a radar pulse and other sensors pick up the reflections, allowing for more subtle and better radar detection. Second, a drone swarm will execute offensive operations through kinetic strikes or assaults utilizing electronic warfare systems. It will be able to strike numerous adversarial targets and assault them in their most vulnerable defense. Behaving in a distracted manner will impede the defender’s response, making it easier for the drone swarm to achieve its objectives without facing significant resistance. If 10 drones assault a target simultaneously and seven are intercepted, three will still succeed in their objective. In the future, it is expected that a substantial drone swarm, which is a large group of drones operating together, would prove to be more efficient and economical than individual humans or UAVs. Third, a drone swarm can be employed for defensive operations, deceiving the adversary with respect to the size and quantity of the aerial combat unit and mitigating their assault. Scharre (2014) [69] illustrates the use of small airborne decoys to deceive adversarial radar systems. He also observed that numerous drones may converge over an adversary’s airstrip to obstruct aircraft from departing. A similar approach could be used to protect a certain area from enemy helicopters or planes that fly over it, but it would become more complicated as the size of the area that needs protection increases.

## 5. Challenges and Future Research Trends

Advancements in machine learning, AI, and drone autonomy are expanding the operational and application potential of drones. The characteristics of navigational autonomy will allow drones to carry out intricate missions without direct human oversight, enhancing security, reliability, and cost-effectiveness in potential uses, including package delivery and monitoring [70]. The advent of 5G and the impending 6G will create novel opportunities for drones by providing faster, more efficient connectivity, and innovative outlooks for the future. Such technology will facilitate high-speed real-time data transfer and autonomous operation of such devices over large distances, allowing more advanced and complex functions to be performed in specified domains. Drones are progressively integrating sophisticated and intricate sensors, including various types of cameras, LIDAR systems, and sensor technologies for the identification of gases and chemical substances [71]. These sensors allow drones to perform activities that include high-resolution 3D mapping, precise industrial inspection, and accurate and efficient ambient control and surveillance. The future of drones holds exciting possibilities and complex challenges. As these nascent technologies become increasingly embedded in our society, it is imperative to meticulously observe their development and guarantee their ethical, safe, and responsible use.

In defense operations leveraging a drone swarm, it is preferable to implement an emergent coordinating model founded on complete autonomy. Nevertheless, although complete autonomy presents distinct advantages for drone swarms, significant concerns are also apparent, such as the potential loss of human oversight, ethical implications in decision-making, and challenges in ensuring reliable communication among drones. These swarms use swarm intelligence, mimicking natural behaviors observed in colonies of bees, ants, or insects, where distributed regulations generate complex cooperative actions. Contemporary swarms incorporate AI and ML to maneuver around barriers such as GPS interference, radio signal interruptions, and challenging hostile environments, ensuring coordinated operations. Command and control methodologies range from predetermined flight trajectories and centralized terrestrial supervision to decentralized control systems that allow drones to engage in dynamic real-time communication [72]. The emergence of drone swarm intelligence signifies a profound transformation in international military tactics [73]. Drone swarms are establishing their prominence in near-future combat operations, either for surveillance, offensive operations, or hybrid operations.

These swarms can exceed conventional air defenses, adjust dynamically, and persist in operations while incurring losses. AI drone swarms are becoming one of the most threatening elements in modern combat as major states compete to deploy them. AI drone swarms are rapidly transforming aerial combat, reallocating power from a limited number of costly platforms to several integrated intelligent systems. As these tools improve, they diminish the obstacles to sophisticated strategic skills while undermining conventional defense methods. From massive countries to smaller states, armed forces are competing to advance both swarm tactics and the requisite countermeasures [74]. The future of aerial dominance will likely hinge not solely on velocity or armament, but on autonomy, scalability, and the capacity for robots to collaborate in real time. Route planning for drone swarms must guarantee not only the ideal global trajectory and minimal time spent on tasks but also the avoidance of obstacles and collisions among individual units during task execution. In the trajectory planning approach, the majority of research treats the drone as a point mass, neglecting its dimensions and payload, hence rendering the modeling method more simplistic and idealized. We must consider the relevant restrictions in the forthcoming modeling procedure [10].

The effective cooperation of drone swarms relies on instantaneous information exchange and communication among agents. In complex contexts, delays in communication, packet loss, and bandwidth constraints may disrupt collaborative operations among agents and diminish mission effectiveness [75]. The integration of developing AI technologies with drone swarm systems is a domain of exploration and progress that is growing in importance. AI techniques, such as ML, DL, and reinforcement learning (RL), are being used in different areas of drone swarm network research to help them learn interactively, make quick decisions, and adjust to changing situations, which improves their performance, flexibility, and dependability [76]. In this case, drones with limited resources could help create lightweight AL/ML frameworks to solve these problems. Moreover, drone swarms want reliable and low-latency networks to facilitate communication, and the integration of such techniques imposes significant expense on such networks. Future research should aim to effectively combine these methods in drone swarm exploration to create a more reliable, interactive, and self-sufficient drone swarm system using different local models [77]. It is essential to develop reliable and economical control algorithms, construct compact and powerful sensors, and enhance the durability and dependability of drones. Future research must concentrate on developing adaptive control techniques capable of responding to dynamic variations in the environment and challenges [78].

Researchers should concentrate on developing power-efficient algorithms for the real-time processing of UAV data, such as aerial imagery, video, and sensing information. It is expected that as UAVs become more advanced, with better features, privacy rules, improved image processing algorithms, cost-effective sensors, longer flight times, and greater carrying capacity, they will be used in many areas, such as studying crops in the field [79]. Vision analysis algorithms for UAVs face several significant challenges, including diverse image orientations, increased overlaps, various sizes, and fluctuating altitudes. The research community must face these problems in forthcoming studies. Additional research efforts should focus on the application of UAVs in robust public safety networks. Future research should focus on public safety communications, health care in crisis scenarios, and the incorporation of blockchain technology in UAVs to improve health monitoring systems. More research is needed to evaluate the impact of poor weather on UAV resilience to guarantee successful mission execution [80]. Current blockchain-enabled UAV applications require authorization or encrypted blockchain networks. These systems are acutely susceptible to threats in the context of multi-UAV networks. Additionally, the increasing number of possible threats, such as violations based on game theory, machine learning attacks, and quantum attacks, makes it essential to strengthen the security of the blockchain [7]. Consequently, research initiatives are required to improve the safety, security, and immutability of private blockchain systems.

## 6. Conclusions

Swarm robotics has had significant advancements, especially with aerial robots. UAV swarms provide a distinctive and effective method for addressing intricate tasks by leveraging the collaborative capabilities of numerous unison robots. AI-driven drone swarms are rapidly transforming the essence of aerial force, reallocating military superiority from costly platforms to synchronized autonomy. AI now enables numerous low-cost drones to function as a cohesive system instead of isolated units. This study provides background information on drone swarms, including the types of drone swarms and the definition of a drone swarm. We have surveyed a wide range of literature in three critical areas, namely, trajectory generation, trajectory prediction, and techniques to counter the drone swarm, which can give different approaches for civilian or combat operations. We discuss some major applications of drone swarms, not only in military warfare but also in protecting civilians, wildlife, and the environment from forest fires caused by climate change. In addition, some research gaps have been identified that can provide future research trends and challenges in this area of study. This article can play a vital role as the backbone of fundamental knowledge for researchers working in this field.

The main research gap is not limited only to the limited integration of realistic and adaptable autonomy across different and changing contexts. Previous studies have improved domains such as coordinated path planning, formation control, and AI-based decision-making, but most of the solutions are either simulation-driven or limited to compact, uniform swarms, lacking resilience under real-world challenges such as communication malfunctions, adversarial constraints, and environmental variance. In addition, the joint optimization of communication, compute, and energy efficiency for large-scale deployments is under-investigated, especially in the case of decentralized systems where resource constraints and network instability have a significant impact on performance. The lack of established frameworks for security, ethical regulation, and scalability is another limitation, particularly as applications are spreading into the defense and civilian sectors. Finally, despite the great potential of AI and RL approaches, there are still issues related to the data quality, explainability, and security of multi-agent learning in partially observable situations. This emphasizes the importance of more flexible, reliable, and field-adaptable swarm intelligence systems.

## Data Availability

No new data were created or analyzed in this study.
